# Supplementary material for: Dopaminergic Input Regulates the Sensitivity of Indirect Pathway Striatal Spiny Neurons to Brain-Derived Neurotrophic Factor
Source: Biology (Basel). 2023 Oct 23;12(10):1360. doi: 10.3390/biology12101360 (PMC10604681; doi:10.3390/biology12101360)
Supplement: Supplementary file 1 [file biology-12-01360-s001.zip › biology-2641020-supplementary.pdf]

Supplemental Information

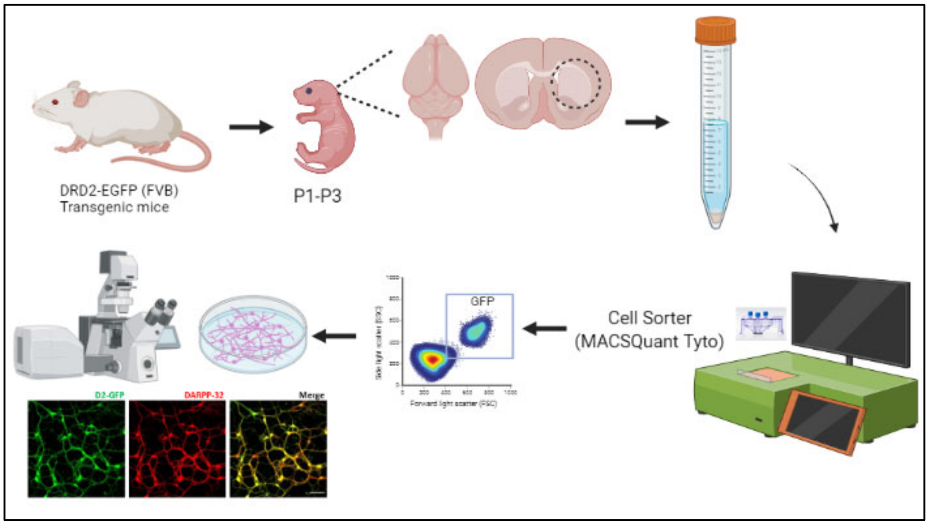

Figure S1. Flow cytometry cell sorting scheme**:** Enrichment of DRD2-positive striatal neurons using fluorescence-based flow cytometry cell sorting.

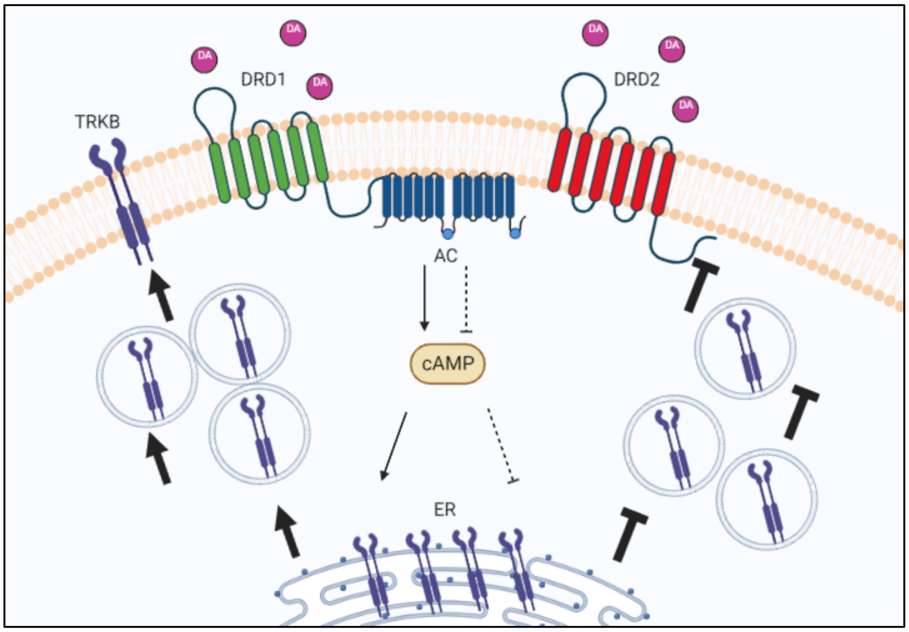

Figure S2. DRD1/ DRD2 - BDNF/TrkB translocation pathways: Activation of DRD1 leads to the facilitation of adenylyl-cyclase activity and an increase in cAMP levels. This increase may cause an export of TrkB from intracellular storage compartments such as the endoplasmic reticulum

(ER) to the cell membrane. Activation of DRD2 in iSPNs leads to the inhibition of adenylyl-cyclase activity and a progressive downregulation of cAMP levels. This decrease in cAMP could prevent TrkB from being translocated to the cell surface.

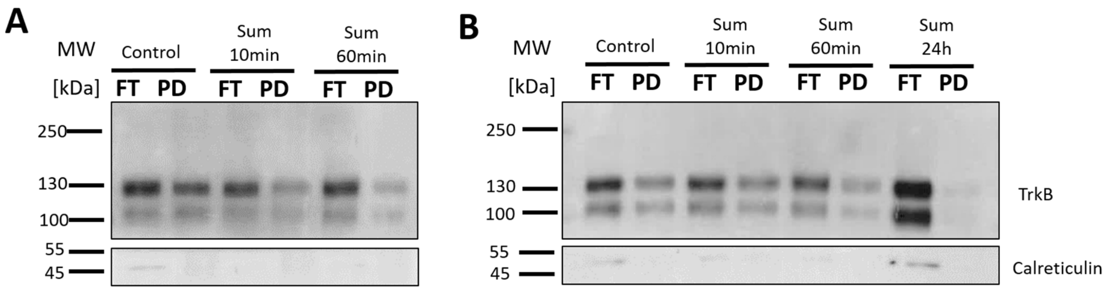

Figure S3. DRD2 activation with Sumanirole decreases TrkB cell surface expression in iSPNs. A-B. Western blot analysis of cell surface biotinylated enriched DRD2-positive neurons at DIV 7-8 stimulated with Sumanirole Maleate (Sum) during different time courses. **A.** Cell surface TrkB (shown as pulldown (PD) of cell surface biotinylated samples) decreases after stimulation of DRD2 neurons for 10 min and 60 min compared to the non-stimulated control. **B.** Longer stimulation of DRD2 with Sumanirole as shown in neurons stimulated for 24h leads to further reduction of cell surface TrkB (PD), while the intracellular fraction shows a higher TrkB concentration (FT). Corresponding quantification of band intensity and statistical analysis are included in Figure 2.

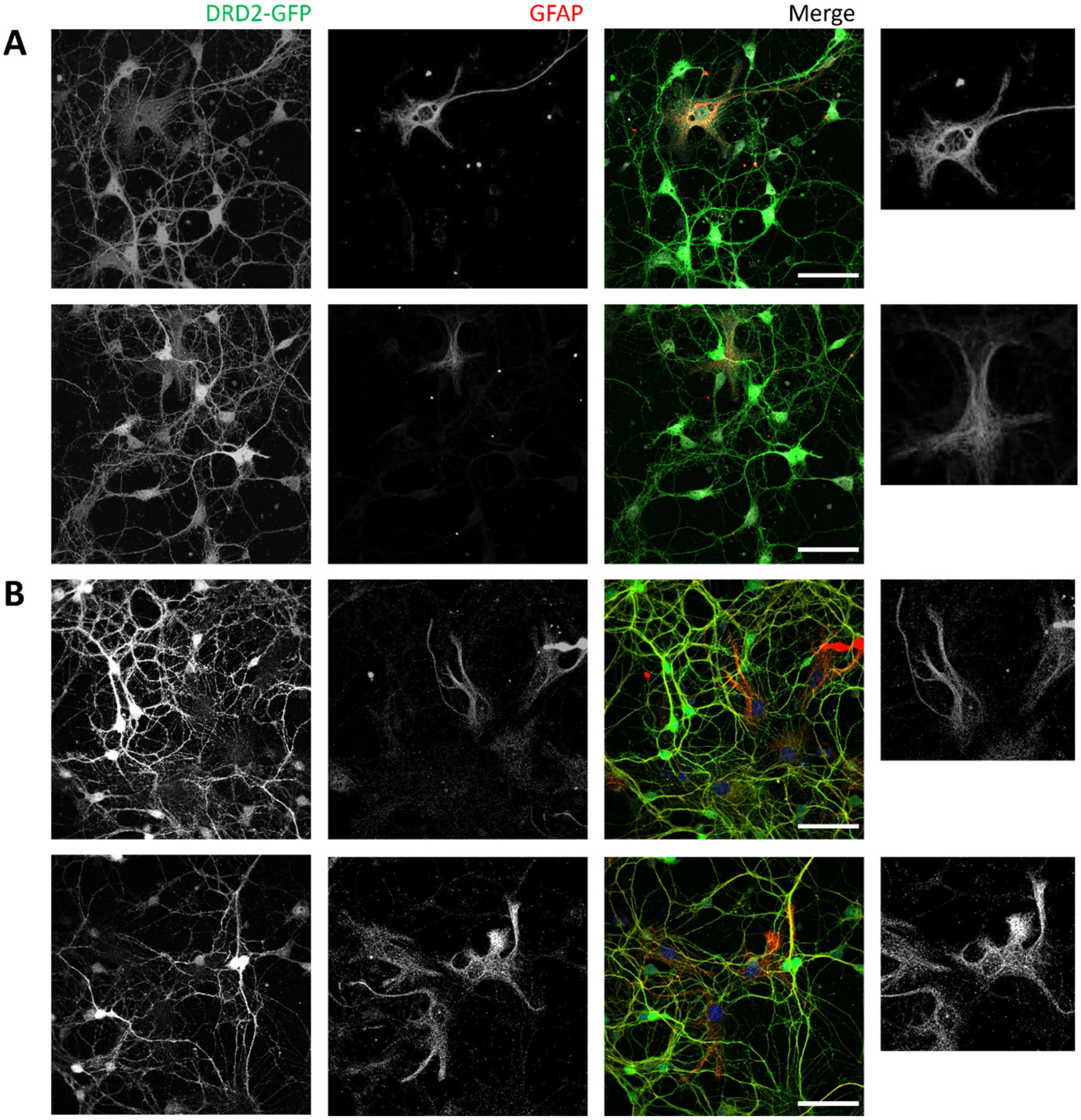

Figure S4. Identification of DRD2-GFP-positive astrocytes. A-B. Immunocytochemistry of DRD2-GFP-positive striatal cells, stained with GFAP as marker for astrocytes, GFP and DAPI. **A.** DRD2-GFP-positive astrocytes at DIV7. Astrocyte number is low, expression of GFAP is also lower as astrocytes are still immature. Magnification of astrocytes shown to the right. **B**. DRD2-GFP-positive astrocytes at DIV13. Astrocyte number increases as well as GFAP expression. Magnification of astrocytes shown to the right.
